# Inequalities in health-related quality of life and the contribution from socioeconomic status: evidence from Tibet, China

**DOI:** 10.1186/s12889-020-08790-7

**Published:** 2020-05-06

**Authors:** JieAnNaMu, Xinpeng Xu, Hua You, Hai Gu, Jinghong Gu, Xiaolu Li, Nan Cui, Yun Kou

**Affiliations:** 1 Center for Health Policy and Management Studies, Nanjing University, Nanjing, China; 2 Department of Social Medicine and Health Education, School of Public Health, Nanjing Medical University, Nanjing, China; 3 Nanjing Foreign Language School, Nanjing, China; 4 Department of Otolaryngology, The First Affiliated Hospital of Nanjing Medical University, Nanjing, China

**Keywords:** Socioeconomic status, Health related quality of life, Health inequality, Tibet, China

## Abstract

**Background:**

This study aimed to understand the association between socioeconomic status (SES) and Health Related Quality of Life (HRQoL) and the contribution of SES to health inequality among Tibetans of agricultural and pastoral areas (APA) in Tibet, China.

**Methods:**

The data were from Health Survey of Tibetans in APA conducted in 2014. A total of 816 respondents were enrolled for the analysis Multiple linear regression was employed to examine the relationship between SES and HRQoL. Concentration index (CI) was used to measure the degree of health inequality and a Wagstaff-type CI decomposition method was applied to measure the contribution of SES to inequality.

**Results:**

SES had significant association with HRQoL among the Tibetans in APA. The high SES group was more likely to have a higher Eq-5d index (0.77 vs. 0.67, *P* < 0.001) and VAS (72.94 vs. 62.41, *P* < 0.001) than the low SES group. The Concentration index of the Eq-5d index and VAS for total sample was 0.022 and 0.026 respectively, indicating a slight pro-rich inequality among this population. The decomposition analyses showed the SES is the main contributor to health inequality and contributed 45.50 and 41.39% to inequality for the Eq-5d index and VAS, respectively.

**Conclusion:**

The results showed SES is positively associated with HRQoL among Tibetans in APA. There was a slight pro-rich inequality in the health of the participants and most health inequality was attributable to SES. This study is helpful in gaining an insight into the HRQoL, health inequality and the relationship between SES and health inequality among Tibetans of APA in China.

## Background

This study investigated Tibetans living in agricultural and pastoral areas (APA) in Tibet, China. Tibet is located on the Qinghai-Tibet plateau in southwest China, and its social and economic development level ranks relatively low in China’s provinces [1]. Most of the population in Tibet is comprised of farmers and herders, who are scattered in remote rural areas. The education level of the population there is relatively low, and their limited source of income mainly depends on agricultural production [2]. In addition, the health risks faced by the population in this area are higher than those in low-altitude areas. Tibetan of APA living in high-altitude areas are faced with poor transportation and communication conditions, and low access to medical services [3]. Some particularities that make the investigation difficult in this group include factors such as region, ethnicity, and lifestyle. Therefore, there is a lack of previous literature on the health status and quality of life of this population. Studies investigating the relationship between socioeconomic status and health status among this population are scarce as well.

Socioeconomic status (SES) which commonly measured by education, income and occupation is an overall measure of the economic and social status of individuals or families relative to others [4]. It has generally been associated with differences in health. A large number of literatures examining the relationship and its stabilization between SES and health [5]. Most studies have agreed that socioeconomic status was the most important determinant of an individual’s health [6–8], since it can affect individual health through a variety of mechanisms. People with low SES are more likely to have unhealthy habits and face higher socio-economic pressure, and SES is also closely correlated with the quantity and quality of health care services available. Despite differences in circumstances and approaches to measuring and analyzing, studies have consistently displayed that people with low SES were more likely to have worse health status [6, 9, 10]. Arguably the same patterns ought to be found in a homogeneous group such as the Tibetans in APA.

On the other hand, the relationship between SES and Health inequality have been discussed extensively, which has achieved a series of important results [7, 11–13]. The monographs of Marmot and Wilkinson (2005), and Bartley (2016) consistently showed that socioeconomic status plays an important role in health and health inequality. Some empirical studies supported similar conclusions. Previous studies have used education and income to measure SES, and discussed its relationship with health inequality [14]. Mackenbach et al. (2008) found that there were differences in the level of health inequality associated with socioeconomic status of European countries [13]. Studies have shown that health inequalities are widespread in many countries, and social class differences in mortality are increasing [13]. Also widening income inequality would do harm to people’s health status [15], and reduced income inequality offered better population health [16]. Analogously, Singh’s research in India found that income and education were the main contributors to health inequality [17]. However, it is not clear whether the conclusions previously reached about health inequality and its determinants in general are consistent in Tibetans of APA.

Health-related quality of life (HRQoL) is an important indicator to measure health status comprehensively and is increasingly used to measure the health inequality among different social groups [18]. As it is directly or indirectly related to a variety of diseases, SES is also regarded as an important factor in determining an individual’s quality of life [19]. In the past decade, SES and its relationship with quality of life has become an important research direction in the field of health care. There are some studies focusing on the correlation between SES and HRQoL, and similar conclusions with the relationship between SES and health are drawn. Low SES is associated with poor HRQoL, even after other confounding factors are adjusted [20–22]. This study performed a population-based study to measure the HRQoL and inequality of the residents in the agricultural and pastoral areas of Tibet, and to understand the correlation between SES and HRQoL in this population.

## Methods

### Data and sampling

**Fig. 1:**
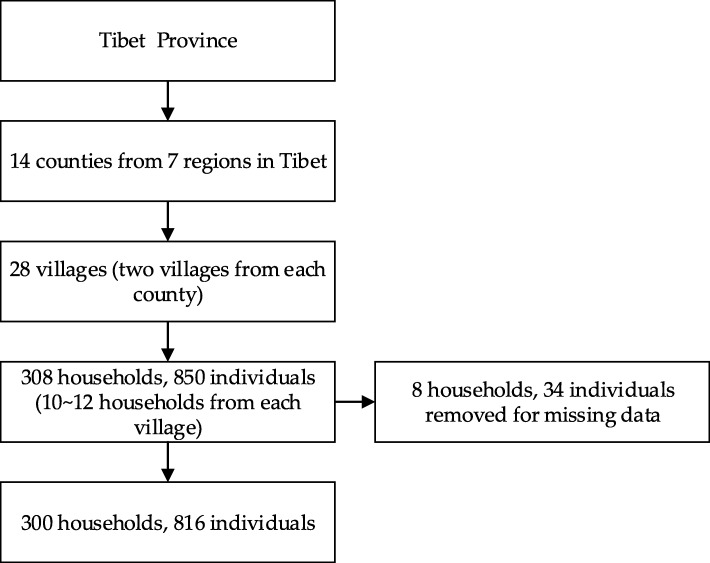
Flow diagram of survey

The data used in this study were derived from Health Survey of Tibetans in APA conducted in 2014. The survey aimed to study the health status and its influencing factors among Tibetans in APA. Some local grass-root health workers were recruited as investigators and attended at the trainings conducted by researchers from Nanjing University and Nanjing Medical University. Of the more than three million people in the Tibet autonomous region, more than 2.5 million are farmers and herders. The survey adopted a multi-stage stratified random sampling strategy based on altitude, infrastructure, and social and economic development. First, counties were randomly selected from seven regions in Tibet (i.e., Lhasa, Nyingchi, Shannan, Xigaze, Qamdo, Naqu, Ali). Second, the administrative villages in each county were randomly selected. Finally, 10 to 12 households were randomly drawn from each village. A total of 308 households comprising 850 individuals from 28 villages in 14 counties were chosen to participate in the survey (Fig. 1).

Participants with missing values on key variables were removed from the sample (34 questionnaires were excluded, accounting for 4%), and a total of 816 valid responses were included in the analysis sample, accounting for 96%.

### Measurement of HRQoL

Eq-5d-3 L was employed to measure the HRQoL of respondents. The equivalence between the Chinese and English version of this scale has been proven [23]. Several studies have also demonstrated its reliability and validity [24, 25]. The scale consists of two parts: a five-dimensional questionnaire and a Visual Analog Scale (VAS). The questionnaire includes mobility, self-care, usual activities, pain/discomfort, and anxiety/depression. Each dimension contains three response levels (1 = no problems, 2 = some/moderate problems, and 3 = extreme problems). The questionnaire could measure 243 possible combinations of health conditions. We employed the Japan Time-Trade Off (TTO) to convert the five dimensions into an index ranging from − 0.11 to 1.00 [26]. TTO is a widely-used method to convert responses to the EQ. 5D scale into specific HRQoL index. The VAS is a standard 0–100 vertical visual analogue scale (like a thermometer from 0 to 100) in order to record an individual’s rating for his/her HRQoL [27]. A respondent having a higher index or VAS score is healthier than others. The Cronbach’s *α* of Eq-5d index was 0.867.

### Measurement of socioeconomic status (SES)

Education, income, and housing conditions were used to define the SES variables. Several studies have used variables such as education, income, and occupation to represent individuals’ socioeconomic status [4, 6, 8, 28–30]. However, there is no difference in occupation since the subjects of our study are farmers in agricultural and pastoral areas. We added housing conditions as one of the main bases for SES classification within the population. The housing conditions used in this study are mainly based on the housing materials and housing facilities of the respondents, including the main source of cooking fuel, type of drink water, toilet facilities, and type of accommodation. The responses are dichotomized as reporting any poor living conditions versus none of the poor conditions. All respondents were asked to report their education level, which was divided into two categories, including illiterate, elementary and above. Given the possible effects of economic scale on household income, we employed the equivalized per capita income (eqpcinc) to represent individual economic condition [31–33]. The calculation formula of eqpcinc is as following Eq. (1), in which *household income* represents the annual actual household income, and *family size* is the actual number of family members.

1 $$ eqpcinc=\frac{household\ income}{{\left( family\ \mathrm{size}\right)}^{0.56}} $$

We generated the dummy income-based group variable based on the median of eqpcinc, representing whether the respondent is relatively wealthy or poor. In addition, poor housing conditions was used as another indicator of socioeconomic status.

We define the SES as a dummy variable. When SES is equal to zero, it indicated individuals classes as illiterate, in the low-income group, and with poor housing conditions. SES is equal to 1 under other values of these three variables, representing high socioeconomic status.

### Covariates

Previous studies have shown that factors associated with HRQoL include individual characteristics, health-related behaviors, access to medical care, chronic disease [34–36]. Accordingly, this study contains these four types of covariates to be taken as confounders. The first category describes individual demographic characteristics, including age, gender, and marital status. The second category describes health-related behaviors, including smoking (the respondent has ever smoked or is a smoker now), brush teeth every day (whether the respondent brushes his or her teeth every day), and medical examination (whether the respondent participated in medical examinations during the last year). The third category was distance (whether the respondent lives at least 3 km from the nearest medical facility), representing the access to medical care. The fourth category was chronic disease (whether the respondent has been diagnosed with any chronic disease).

### Statistical analysis

Multiple linear regressions were employed to examine the relationship between SES and HRQoL. The dependent variables included the Eq-5d index and VAS. SES was the core independent variable in regression models.

The concentration index (CI) is widely used to measure health inequalities associated with socioeconomic status [32, 33, 37, 38]. The CI is defined as twice the area between the concentration curve and the line of equality. In our study, heath inequality was calculated as follows:

2 $$ CI=\frac{2}{n\cdot \mu}\sum \limits_{i=1}^n{HRQoL}_i{R}_i-1 $$

Where *HRQoL*_*i*_ is the Eq-5d index or VAS score of the *i*^th^ individual, *n* is the number of observations, *μ* is the average of Eq-5d index or VAS. *R*_*i*_ is the *i*^th^ individual’s rank in terms of *eqpcinc*. The range of CI is [− 1,1]. When CI is 0, the concentration curve is an equality line of 45 degrees, and there is no inequality. When CI is positive, the concentration curve is below the equality line, indicating the existence of pro-rich inequality. When CI is negative, the concentration curve is above the equality line, indicating the existence of pro-poor inequality. A higher absolute CI means a higher level of health inequality. This study adopted a Wagstaff-type CI decomposition method to analyze the contributions of different factors to health inequality [39]. The calculation process is as follows:

First, the following linear regression model is established:

3 $$ {HRQoL}_i=\alpha +{\sum}_k{\beta}_k{x}_{ki}+{\varepsilon}_i $$

Where *α* represents the intercept, *x*_1_, ⋯, *x*_*k*_ represent the *k* independent variables, *β*_1_, ⋯, *β*_*k*_ represent the corresponding coefficients, and *ε*_*i*_ represents the error term.

Secondly, the CI of HRQoL can be rewritten based on the above model as follows:

4 $$ CI={\sum}_k\left({\beta}_k{\tilde{x}}_k/\mu \right){C}_k+{GC}_{\varepsilon }/\mu $$

where $\tilde{x}{}_k$ represents the means of the *k*^th^ independent variable, ${GC}_{\varepsilon }=\frac{2}{n}\sum \limits_{i=1}^n{\varepsilon}_i{R}_i$, *C*_*k*_ represents the CI of the *k*^th^ variable based on the same calculation formula of CI (Eq.(1)) for HRQoL. Eq. (3) indicates that the CI of HRQoL consists of deterministic and residual components. $\left({\beta}_k{\tilde{x}}_k/\mu \right){C}_k$ is the contribution of the *k*^th^ determinant of health inequality, and the contribution rate is $\frac{\left({\beta}_k{\tilde{x}}_k/\mu \right){C}_k}{CI}\times 100\%$.

The Stata 15.1 (StataCorp., College station, Texas) were used for data analysis in the study.

## Results

### Characteristics of the study population

Table 1 describes the basic characteristics of the study population. The average age of the respondents was 38.74 years (SD = 18.82), 426 (52.21%) were female, and 33.7% were not married. For socioeconomic variables, 62.87% were illiterate, 66.67% had poor housing conditions, and the annual equivalized per capita income was 71,500 Yuan (10,600 dollars). A total of 40.07% of the respondents lived on less than $1.90 a day, which has been suggested as the international poverty line by the World Bank [40, 41]. The average score of Eq-5d index and VAS was 0.74 and 69.9, respectively.

**Table 1:** Description of variables (*N* = 816)

Variables	Total sample (*N* = 816)		Female (*N* = 426)		Male (*N* = 390)	
	N/mean	%/SD	N/mean	%/SD	N/mean	%/SD
Age	38.74	18.82	38.89	18.97	38.57	18.68
Gender						
Female	426	52.21				
Male	390	47.79				
Marital Status						
Unmarried	275	33.70	143	33.57	132	33.85
Married	541	66.30	283	66.43	258	66.15
Smoking						
No	364	44.61	214	50.23	150	38.46
Yes	452	55.39	212	49.77	240	61.54
Brush teeth every day						
No	570	69.85	290	68.08	280	71.79
Yes	246	30.15	136	31.92	110	28.21
Medical examination						
No	588	72.06	302	70.89	286	73.33
Yes	228	27.94	124	29.11	104	26.67
Distance						
Within three kilometers	322	39.46	179	42.02	143	36.67
Three kilometres away	494	60.54	247	57.98	247	63.33
Chronic diseases						
Without	590	72.30	305	71.60	285	73.08
With	226	27.70	121	28.40	105	26.92
Socioeconomic Status						
Low	236	28.92	138	32.39	98	25.13
High	580	71.08	288	67.61	292	74.87
Educational Level						
Illiteracy	513	62.87	298	69.95	215	55.13
Elementary and above	303	37.13	128	30.05	175	44.87
Poor housing conditions						
Yes	544	66.67	297	69.72	247	63.33
No	272	33.33	129	30.28	143	36.67
Eqpcinc (thousand Yuan)	7.15	8.62	6.88	8.47	7.44	8.78
Income-based group						
Quintile1 (poor)	408	50.00	218	51.17	190	48.72
Quintile2 (rich)	408	50.00	208	48.83	200	51.28
Below the international poverty line ^a^						
No	489	59.93	252	59.15	237	60.77
Yes	327	40.07	174	40.85	153	39.23
Eq-5d index	0.74	0.18	0.73	0.18	0.75	0.18
VAS	69.90	19.33	69.02	18.95	70.86	19.72

^a^ The variable is defined if the per capita income of an individual is below the international poverty line ($ 1.9 a day). Since the 2011 PPPs for Rural China is 3.04, the international poverty line for China rural residents is 1.9*3.04*365 = 2108.24 Yuan. If the per capita income of an individual was less than 2108.24 Yuan, the individual lived below the international poverty line

### HRQoL among different SES groups

Table 2 described the average Eq-5d scores of different groups in the total sample and gender samples. For the total sample, the high SES group was more likely to have a higher Eq-5d index (0.77 vs. 0.67, *P* < 0.001) and VAS (72.94 vs. 62.41, *P* < 0.001) than the low SES group. Specifically, the individuals with formal education, relatively higher income, and non-poor housing conditions were more likely to have higher Eq-5d index and VAS than their corresponding comparison groups. The results of Eq-5d index and VAS for different SES variables are similar in gender subsamples.

**Table 2:** The HRQoL among different population group

Variables	Total sample (*N* = 816)				Female (*N* = 426)		Male (*N* = 390)	
	Eq-5d index		VAS		Eq-5d index	VAS	Eq-5d index	VAS
	Mean/SD	t-test^a^	Mean/SD	t-test^a^	Mean/SD	Mean/SD	Mean/SD	Mean/SD
Socioeconomic Status								
Low	0.67/0.20	7.27^***^	62.41/20.08	7.28^***^	0.66/0.19	62.16/19.32	0.67/0.21	62.77/21.20
High	0.77/0.16		72.94/18.18		0.76/0.16	72.31/17.89	0.77/0.17	73.58/18.46
Educational Level								
Illiteracy	0.68/0.18	12.97^***^	63.57/19.34	13.43^***^	0.68/0.18	63.51/18.89	0.68/0.19	63.66/19.99
Elementary and above	0.83/0.12		80.61/13.83		0.84/0.10	81.84/11.41	0.82/0.14	79.71/15.33
Poor housing conditions								
Yes	0.71/0.19	6.16^***^	66.92/19.77	6.38^***^	0.71/0.18	66.41/19.44	0.72/0.19	67.53/20.18
No	0.79/0.15		75.86/16.95		0.78/0.15	75.03/16.31	0.80/0.15	76.61/17.53
Income-based group								
Quintile1 (poor)	0.72/0.19	3.07^**^	67.80/19.74	3.11^**^	0.71/0.18	67.10/19.04	0.73/0.19	68.61/20.53
Quintile2 (rich)	0.76/0.17		72.00/18.71		0.75/0.17	71.03/18.69	0.76/0.17	73.00/18.72

*P < 0.05,**P < 0.01,***P < 0.001

^a^ The t test was employed to examine whether there was a significant difference in the mean between the two groups of a variable

### The relationship between SES and HRQoL

The results of multiple linear regression showed that SES was significantly associated with Eq-5d index (Table 3)/VAS (Table 4). For the total sample, the low SES group has a lower Eq-5d index (*P* = 0.002) and VAS (*P* = 0.002) than others. For females and males, the results of the association between SES and the Eq-5d were similar for the index (*P* = 0.02, *P* = 0.035) and VAS (*P* = 0.03, *P* = 0.03).

**Table 3:** Factors influenced Eq-5d index (Multiple linear regression model)

Variables	Total sample (*N* = 816)		Female (*N* = 426)		Male (*N* = 390)	
	Coef. (β_**i**_)	95% Conf. interval	Coef. (β_**i**_)	95% Conf. interval	Coef. (β_**i**_)	95% Conf. interval
Socioeconomic Status (Ref: low)	0.05^**^	(0.02,0.08)	0.05^*^	(0.006,0.09)	0.05^*^	(0.004,0.10)
Age	−0.004^***^	(−0.005,−0.004)	-0.004^***^	(−0.005,-0.003)	−0.005^***^	(−0.006,−0.004)
Gender (Ref: female)	0.008	(−0.01,0.03)				
Marital status (Ref: unmarried)	0.05^***^	(0.02,0.07)	0.03	(− 0.006,0.06)	0.07^***^	(0.04,0.11)
Smoking (Ref: no)	0.04^**^	(0.02,0.07)	0.03^*^	(0.0005,0.07)	0.05^**^	(0.02,0.08)
Brush teeth every day (Ref: no)	0.009	(− 0.01,0.03)	0.02	(−0.02,0.05)	0.005	(−0.03,0.04)
Medical examination (Ref: no)	0.02	(−0.003,0.05)	0.03	(−0.01,0.06)	0.02	(−0.02,0.06)
Distance (Ref: within three kilometers)	-0.004	(−0.03,0.02)	−0.0002	(− 0.03,0.03)	−0.01	(− 0.04,0.02)
Chronic disease (Ref: without)	−0.02	(− 0.05,0.004)	−0.01	(− 0.05,0.03)	−0.03	(− 0.07,0.003)
Constant	0.81^***^	(0.77,0.85)	0.81^***^	(0.76,0.86)	0.82^***^	(0.76,0.88)

*P < 0.05,**P < 0.01,***P < 0.001

**Table 4:** Factors influenced VAS (Multiple linear regression model)

Variables	Total sample (N = 816)		Female (N = 426)		Male (N = 390)	
	Coef. (β_i_)	95% Conf. interval	Coef. (β_i_)	95% Conf. interval	Coef. (β_i_)	95% Conf. interval
Socioeconomic Status (Ref: low)	5.01^**^	(1.88,8.15)	4.64^*^	(0.42,8.86)	5.36^*^	(0.60,10.13)
Age	−0.47^***^	(−0.54,-0.40)	− 0.43^***^	(− 0.53,-0.33)	−0.53^***^	(− 0.63,-0.43)
Gender (Ref: female)	0.85	(−1.48,3.17)				
Marital status (Ref: unmarried)	5.24^***^	(2.81,7.66)	2.73	(−0.63,6.08)	8.50^***^	(4.93,12.07)
Smoking (Ref: no)	5.00^***^	(2.45,7.55)	4.28^*^	(0.73,7.83)	5.76^**^	(2.10,9.42)
Brush teeth every day (Ref: no)	0.77	(− 1.79,3.32)	1.61	(−1.91,5.13)	0.32	(−3.44,4.07)
Medical examination (Ref: no)	3.78^**^	(0.97,6.58)	4.00^*^	(0.09,7.91)	3.29	(−0.72,7.30)
Distance (Ref: within three kilometers)	−0.19	(−2.58,2.20)	− 0.08	(−3.32,3.17)	−0.68	(−4.21,2.86)
Chronic disease (Ref: without)	−2.48	(−5.29,0.32)	−1.08	(−4.89,2.74)	−4.04	(−8.23,0.15)
Constant	77.33^***^	(73.21,81.46)	77.28^***^	(71.66,82.89)	78.69^***^	(72.62,84.76)

*P < 0.05, **P < 0.01, ***P < 0.001

### Health inequality and its decomposition

Table 5 shows the CI of Eq-5d index/VAS in different groups. For the total sample, the CI of the Eq-5d index and VAS were 0.022 and 0.026, while they were 0.023 and 0.027 for females, and 0.021 and 0.025 for males, respectively.

**Table 5:** Concentration index of Eq-5d index and VAS

Variables	Total		Female		Male	
	CI	95% CI	CI	95% CI	CI	95% CI
Eq-5d index	0.022	(0.012,0.032)	0.023	(0.010,0.036)	0.021	(0.006,0.035)
VAS	0.026	(0.015,0.037)	0.027	(0.012,0.042)	0.025	(0.009,0.041)

Table 6 describes the CI decomposition of the health inequality in the total sample and gender subgroups. For the total sample, SES contributed 45.50 and 41.39% to health inequality for the Eq-5d index and VAS, respectively. For females, the contribution of SES to health inequality was 44.29% for the Eq-5d index and 39.39% for VAS. Similarly, for males, 46.37 and 43.27% of the health inequality for the Eq-5d index and VAS was attributable to SES, respectively.

**Table 6:** Contributions of Socioeconomic Status and other factors for health inequality (%)

	Eq-5d index			VAS		
	Total	Female	Male	Total	Female	Male
Socioeconomic Status	45.50	44.29	46.37	41.39	39.34	43.27
Other factors	31.44	25.86	38.12	34.64	29.02	40.90
Total	76.94	70.15	84.48	76.03	68.36	84.17

## Discussion

This study revealed the health status and health inequality of residents in the agricultural and pastoral areas of Tibet based on HRQoL, analyzed the relationship between SES and HRQoL among Tibetans in APA, and estimated the contribution of SES on the health inequality of the population.

The results revealed the relatively low HRQoL among Tibetans in APA in China. In western China, rural residents in Shaanxi province scored an Eq-5d index of 0.95 [42], higher than HRQoL of the participants of this study in Tibet. Compared to the other population in Tibet, Tibetans in APA also had lower HRQoL. A survey of medical staff in Tibet showed that the average score of the Eq-5d index was 0.79, and VAS was 75.02 [43], which is higher than the HRQoL in this study.

The findings of this study showed that low SES was significantly associated with low HRQoL. Consistent with this study, a recent study of the general population revealed that high SES is positively associated with the quality of life of Chinese people [44]. Similar results were found in studies of ethnic minorities in China; a study of Dai residents in Yunnan province showed that the higher their socioeconomic status, the higher their life quality score [45]. Another survey among the residents of the Hui ethnic minority group showed that socioeconomic status affects health level, and the influence was more significant in rural areas than in urban areas [46]. The conclusions drawn in this study also corroborate the findings of other countries [47, 48]. SES determines people’s living and working environment and determines accessibility to a variety of health products and services [34]. Furthermore, SES affects people’s psychological state and cognition of the world around them [49, 50]. These physical and psychological factors, in turn, influence how people behave and the probability of exposure to various risk factors that affect their health [51].

The results revealed that there was pro-rich inequality in the health of Tibetans in APA, but the degree of inequality is relatively low. Similar conclusions have been drawn from previous studies in China [52], and in urban and rural areas in this country [53, 54]. Studies targeting specific groups, such as the elderly and rural residents in western China, also revealed pro-rich health inequality [55, 56].

SES contributed to inequality in over 40% of all factors considered in this study. Most studies have reached consistent conclusions on the contribution of SES to health inequality. Income is the main factor affecting health inequality [57]. For both urban and rural residents, income was the most significant contributing factor to health inequality [53, 54, 58] and widening income inequality increased healthy inequality [15, 59, 60]. Other socioeconomic variables such as region, education level, and occupation are also major factors affecting health inequality [61, 62]. Studies of specific populations, such as the elderly and migrant populations, have shown similar results [63, 64]. The impact of SES on health inequality is achieved through several indicators, among which education, occupation, income, housing conditions, household registration, and other relevant demographic factors all have impacts on health inequality. In this study, education, income, and housing conditions were included in the measurement of SES differences because of the characteristics of the participants. Tibetans in APA are generally poorly educated and a high proportion are illiterate. Schooling has obvious influence on health cognition and health literacy [50, 65], as well as health-related lifestyle [66–68]. This study indicated that the relationship between SES and HRQoL among Tibetans deserves strong attention. The implementation of some strategies that help to improve SES can also improve HRQoL of Tibetans in APA to a large extent, such as strengthening the elementary education, the publicity and education regarding public health, and conducting extensive health education and healthy lifestyle guidance. Income and housing conditions also reflected the SES situation of Tibetans in APA. The agricultural and pastoral areas in Tibet are areas with high concentrations of poverty [2]. Our study found that 40.07% of the subjects lived below the international poverty line. To help poor families in agricultural and pastoral areas, the government should actively implement supportive measures. Effective poverty alleviation policies aimed at local residents would be helpful to improve the quality of life and overall health of Tibetans in APA. Strengthening public infrastructure such as reconstructing water supply and lavatory would also be helpful to decrease the health risks the local residents are faced with.

Our study has some limitations. First, the cross-sectional data used in this study can only explain the correlation between SES and HRQoL, but fail to examine the causal relationship. The inference of the causal relationship between SES and HRQoL among Tibetans in APA needs to be further verified. Second, due to the difficulty of investigation and the availability of the data, the sample size is relatively small. Also since the sample is not strictly proportional to the population of different counties and villages, the sample representation may be relatively weak, which may result in selection bias. Third, in some studies, subjective social status has shown a better correlation with health than objective social status. A longitudinal study of Britain also showed that subjective social status was a better predictor of health than income and education [69]. The combination of some subjective and objective indicators should be considered to measure socioeconomic status in future studies.

## Conclusions

This study revealed a slight pro-rich inequality in the health of Tibetans in agricultural and pastoral areas in China. SES was found to be the main contributing factor to health inequality, and low SES is associated with relatively poor quality of life among Tibetans in APA. This particular group of Tibetans, especially poor people without formal education, deserves more attention. Targeted policies and strategies need to be strengthened, including education improvement and poverty alleviation.

## Data Availability

Data are available upon reasonable request from the corresponding author or Center for health policy and management studies, Nanjing University. Email: youhua98@163.com

## Abbreviations

SES: Socioeconomic status
HRQoL: Health-related quality of life
APA: Agricultural and pastoral areas
CI: Concentration Index
VAS: Visual Analog Scale
TTO: Time-Trade Off
